# Supplementary material for: Bioprospecting of Targeted Phenolic Compounds of Dictyota dichotoma, Gongolaria barbata, Ericaria amentacea, Sargassum hornschuchii and Ellisolandia elongata from the Adriatic Sea Extracted by Two Green Methods
Source: Mar Drugs. 2023 Jan 29;21(2):97. doi: 10.3390/md21020097 (PMC9962685; doi:10.3390/md21020097)
Supplement: Supplementary file 1 [file marinedrugs-21-00097-s001.zip › marinedrugs-2137308-supplementary.pdf]

Supplementary material

Bioprospecting of targeted phenolic compounds of *Dictyota dichotoma*, *Cystoseira barbata*, *Ericaria amentacea*, *Sargassum hornschuchii* and *Ellisolandia elongata* from the Adriatic Sea extracted by two green methods

Aly Castillo [1,2], Maria Celeiro [1,2], Marta Lores [2], Kristina Grgić [3], Marija Banožić [3,4], Igor Jerković [5,*] and Stela Jokić [3,*]

1 CRETUS, Department of Analytical Chemistry, Nutrition and Food Science, Universidade de Santiago de Compostela, E-15782 Santiago de Compostela, Spain
2 LIDSA, DepartmeSnt of Analytical Chemistry, Nutrition and Food Science, Faculty of Chemistry, Universidade de Santiago de Compostela, E-15782 Santiago de Compostela, Spain
3 Department of Process Engineering, Faculty of Food Technology Osijek, University of Josip Juraj Strossmayer in Osijek, Franje Kuhača 18, 31000 Osijek, Croatia
4 Faculty of Agriculture and Food Technology, University of Mostar, Biskupa Čule bb, 88000 Mostar, Bosnia and Herzegovina
5 Department of Organic Chemistry, Faculty of Chemistry and Technology, University of Split, Ruđera Boškovića 35, 21000 Split, Croatia
* Correspondence: igor@ktf-split.hr (I.J.); stela.jokic@ptfos.hr (S.J.)

Table S1. Target polyphenols. CAS number, molecular mass, retention time and MS/MS transitions.

Polyphenols	CAS	Molecular mass (g mol^{-1})	Retention time (min)	MS/MS transitions*		
				Precursor ion	Product ions	Collision energy, eV
Gallic acid	149-91-7	170.1	2.35	169.0	125.0, 153.1	17, 15
2,4,6-trihydroxybenzoic acid	71989-93-0	188.1	3.37	168.9	150.9, 107.0	17, 22
Gallocatechin	970-73-0	306.3	3.80	305.0	175.1, 158.4	19, 12
3,4-dihydroxybenzoic acid	99-50-3	154.1	3.88	152.9	109.0, 91.0, 108.0	17, 19, 23
2,4-dihydroxybenzoic acid	89-86-1	154.1	4.41	153.0	109.0, 65.0, 67.0	16, 19, 23
2,5-dihydroxybenzoic acid	490-79-9		4.44	153.0	109.0, 65.0, 67.0	16, 19, 23
Caftaric acid	67879-58-7	312.2	4.48	310.9	178.9, 310.9, 148.9	17, 14, 14
2,5-dihydroxybenzaldehyde	1194-98-5	138.1	4.63	136.9	81.0, 109.0	21, 18, 14
3,4-dihydroxybenzaldehyde	139-85-5	138.1	4.64	136.9	81.0, 109.04	21, 24, 25
3-hydroxybenzoic acid	99-06-9	138.1	5.15	137.0	93.0, 65.0	17, 27
4-hydroxybenzoic acid	99-96-7	138.1	5.18	137.0	93.0, 65.0	17, 27
Procyanidin B1	20315-25-7	578.5	5.30	577.0	407.0, 288.9, 424.9	26, 25, 26
Catechin	18829-70-4	290.3	5.34	289.0	245.0, 203.1	17, 22
2,6-dihydroxybenzoic acid	303-07-1	154.1	5.54	153.0	109.0, 135.0	17, 16
3,5-dihydroxybenzoic acid	99-10-5	154.1	5.54	153.0	109.0, 135.0	17, 16
3-hydroxybenzaldehyde	100-83-4	122.1	5.67	121.0	93.0, 92.0, 120.0	20, 23, 19
4-hydroxybenzaldehyde	123-08-0	122.1	5.67	121.0	93.0, 92.0, 120.0	20, 23, 19

3,4-dimethoxybenzoic acid	93-07-2	182.0	6.03	182.9	137.0, 106.9	6, 22
Procyanidin B2	29106-49-8	578.5	6.10	577.0	407.0, 288.9, 424.9	26, 25, 26
Chlorogenic acid	327-97-9	354.3	6.12	353.0	191.0, 85.0, 93.0	22, 43, 45
Ampelopsin	27200-12-0	320.25	6.20	319.0	193.1, 301.1, 125.1	15, 13, 27
Caffeic acid	331-39-5	180.2	6.50	178.8	135.0, 134.0	19, 28
Procyanidin C1	37064-30-5	866.8	6.52	577.0	288.3, 407.0, 424.9	26, 25, 26
Epicatechin	35323-91-2	290.3	6.56	289.0	245.0, 203.1	17, 22
Epigallocatechingallate	989-51-5	458.4	6.79	457.1	169.0, 125.0, 305.0	21, 42, 21
Procyanidin A1	12798-56-0	576.5	6.80	577.0	407.0, 288.9, 424.9	26, 25, 26
Gallocatechingallate	84650-60-2	458.4	7.29	457.1	169.0, 125.0, 305.0	21, 42, 21
Procyanidin A2	41743-41-3	576.5	7.32	577.0	287.0, 136.9, 425.0	32, 62, 13
Orientine	28608-75-5	448.4	7.38	477.1	327.1, 357.1	23, 22
7-hydroxycoumarin	93-35-6	162.1	7.80	162.9	107.0, 77.0, 91.0	22, 34, 20
4-hydroxycinnamic acid	501-98-4	164.2	7.89	163.0	119.0, 93.0, 117.0	17, 37, 38
Epicatechingallate	1257-08-5	442.4	7.92	441.1	289.1, 125.0, 169.0	20, 42, 24
Polidatin	27208-80-6	390.4	7.98	389.1	227.1, 185.1, 143.1	22, 38, 48
Taxifolin	480-18-2	304.25	8.06	303.0	285.1, 125.1, 175.1	14, 23, 22
Catechingallate	130405-40-2	442.3	8.12	441.1	289.1, 125.0, 169.0	20, 42, 24
3,4-dimethoxybenzaldehyde	120-14-9	166.2	8.33	167.0	139.0, 108.0, 124.0	13, 21, 18
Trans-ferulic acid	537-98-4	194.2	8.92	192.8	177.9, 133.9	12, 16
4-methoxybenzaldehyde	123-11-5	136.1	9.11	136.9	109.0, 77.0, 94.0	12, 23, 18
Resveratrol	501-36-0	228.07	9.31	227.1	185.1, 115.1, 117.1	21, 47, 20
Robinetin	490-31-3	302.23	9.50	301.2	162.6, 273.0	20, 20
Quercetin-3-glucuronide	22688-79-5	478.4	9.54	479.0	461.5, 302.9	14, 18
Quercetin-3-rutinoside	153-18-4	610.5	9.72	609.1	270.9, 178.8, 300.0	96, 44, 37
Quercetin-3-glucoside	482-35-9	463.4	9.75	465.0	256.9, 302.9	41, 14
Myricetin	529-44-2	318.2	10.09	319.0	153.0, 217.0, 245.0	31, 31, 27
Ellagic acid	476-66-4	302.19	10.21	301.0	145.1, 185.1, 173.1	39, 29, 36
Rosmarinic acid	20283-92-5	360.3	10.37	359.0	161.1, 133.1, 135.1	23, 42, 38
3,4,5-trimethoxycinnamic acid	90-50-6	238.2	11.13	239.0	221.0, 162.9, 190.0	11, 27, 19
Astragalin	480-10-4	448.4	11.16	447.0	255.1, 284.1, 227.1	40, 28, 49
3,5-dimethoxybenzaldehyde	7311-34-4	166.2	11.26	167.1	124.0, 77.0	17, 26
Fisetin	528-48-3	286.2	11.50	285.0	135.0, 163.0	15, 15
Daidzein	486-66-8	254.2	11.53	253.0	133.1, 91.1, 135.1	34, 40, 30
Epsilon-viniferin	62218-08-0	454.5	11.82	455.1	361.1, 349.1, 350.1	20, 20, 20
Quercetine	117-39-5	302.2	11.83	303.0	229.1, 153.0	28, 33
Naringenin	67604-48-2	272.2	11.89	272.0	119.0, 147.0	20, 22
Luteolin	491-70-3	286.2	12.17	285.0	199.0, 175.0, 241.0	20, 22, 20
Kaempferol	520-18-3	286.2	12.35	285.0	184.9, 239.1	30, 35
Isorhamnetin	480-19-3	316.3	12.52	317.0	302.0, 165.0, 257.0	20, 24, 35
Diosmetin	520-34-3	300.26	12.64	299.0	133.1, 63.1, 65.1	46, 48, 54
Apigenin	520-36-5	270.2	12.63	269.0	117.1, 149.1, 151.0	37, 26, 26
Chrysin	480-40-0	254.2	13.24	253.1	143.1, 63.2, 145.1	30, 34, 31

* Underlined MS transition is used for quantification purpose
